# Labial Adhesion with Acute Urinary Retention Secondary to Vaginitis

**DOI:** 10.1155/2014/259072

**Published:** 2014-07-07

**Authors:** Şenol Şentürk, Pelin Üstüner, Gülşah Balık, Mehmet Kağıtcı, Ülkü Mete Ural, Figen Kır Şahin

**Affiliations:** ^1^Department of Obstetrics and Gynecology, Faculty of Medicine, Recep Tayyip Erdoğan University, Islampasa Mahallesi, Sehitler Caddesi, No. 74, Merkez, 53100 Rize, Turkey; ^2^Dermatology Clinic, Rize State Hospital, Merkez, 53100 Rize, Turkey

## Abstract

Labial adhesion occurs most often in infants and girls and is usually associated with low estrogen levels. Labial adhesion in the reproductive age group is extremely rare due to abundance of estrogen. Herein we present a case of almost complete labial adhesion with acute urinary retention in a 21-year-old virgin woman secondary to a probable untreated severe vaginitis.

## 1. Introduction

Labial adhesion is defined as either partial or complete adherence of the labia minora or majora [1]. It occurs most often in infants and girls and is usually associated with low estrogen levels [2]. It rarely occurs in postmenopausal women and is associated with hypoestrogenic state, local inflammatory and irritative conditions, and vulvar dystrophies such as lichen sclerosis [3]. Labial adhesion in the reproductive age group is extremely rare due to abundance of estrogen. The etiology of it is unknown in this age group.

Herein we present a case of almost complete labial adhesion with acute urinary retention in a reproductive aged woman secondary to a probable untreated severe vaginitis.

## 2. Case Report

A 21-year-old virgin woman attended at the gynecological outpatient department of our hospital with acute pelvic pain and bladder distension. She was unable to urinate during the previous 12 hours. She had a history of increasing difficulty of passing urine and pelvic pain during the last menstruation two weeks before. She had no history of surgical intervention and her medical history was insignificant. Her menstrual cycles were regular since menarche at 10 years of age.

**Figure 1:**
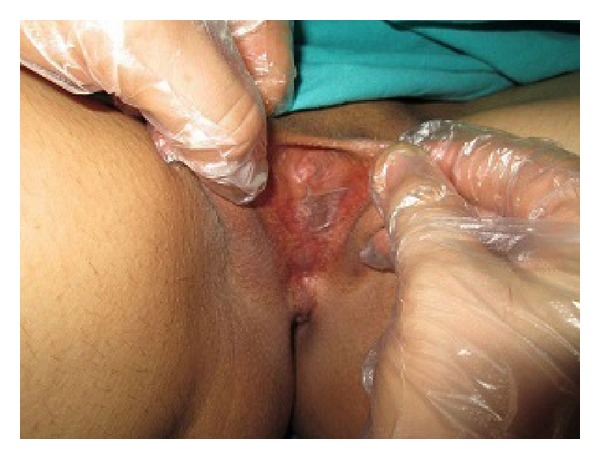
Complete fusion of the labia minora with obliteration of the vaginal introitus and urethral meatus.

On examination, she had normal secondary sexual characteristics. Examination of the genital area showed almost complete adhesion of the labia minora and bladder outlet obstruction (Figure 1). Pelvic ultrasound and laboratory work-up were normal. Dermatological examination of the vulva was unremarkable. Upon further questioning she noted that she had vaginal pain, burning, and profuse yellow vaginal discharge three weeks before. Her family doctor indicated that the vulvar vestibule was thinned, sensitive, erythematous, and edematous as a result of irritation from the discharge. She was referred to a gynecologist but she refused to go and to take any medication.

**Figure 2:**
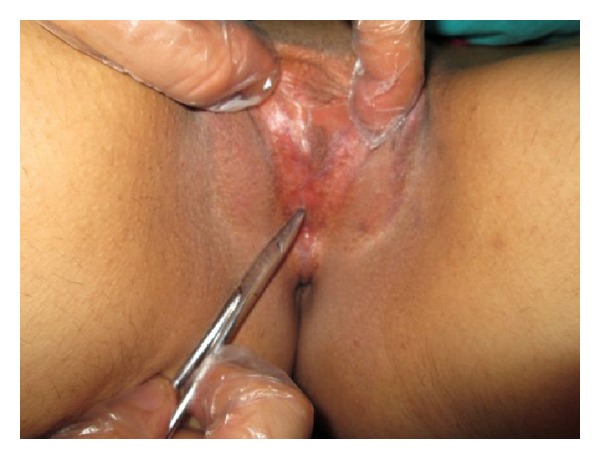
First surgical intervention.

**Figure 3:**
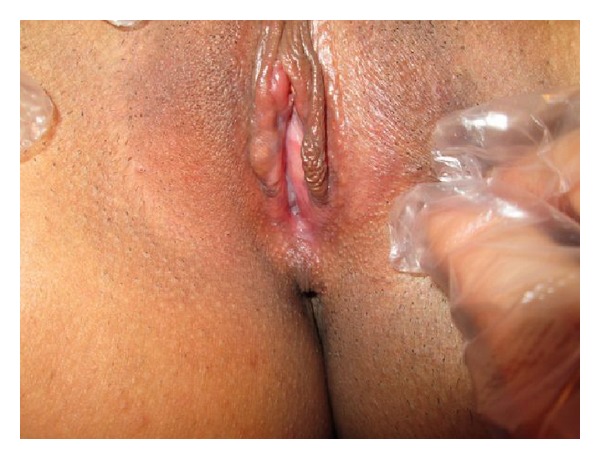
Postoperative 15th day view on patient.

After discussion of therapeutic options and informed consent was taken, a small space was obtained from the weakest point of the adhesion with a thin clamp (Figure 2). Topical therapy with prednisolone, estrogen, and 2% clindamycin cream was administrated on the labia minora for three days. The labial space was gradually increased with local anesthesia and a thin clamp for this time. After three days we could not manage to achieve the desired result, so the patient underwent surgical intervention with general anesthesia. The labia minora were completely separated from the translucent line of the labial adhesion area. Topical therapy with prednisolone, estrogen, and 2% clindamycin cream was administered for an additional one week after surgery. The postoperative follow-up was uneventful and the patient was discharged on the postoperative 4th day. The punch biopsy of the adhesion area, saline microscopy of vaginal discharge, and culture results were unremarkable. The patient had no complaint at the postoperative 15th day and 6th month control (Figure 3).

## 3. Discussion

The exact causes of labial adhesions are uncertain in reproductive aged women. Labial adhesions have been found secondary to female circumcision, herpes simplex, dermatological conditions, caustic vaginitis, local trauma, and vaginal laceration following childbirth [1, 4–7].

Inflammation and irritation secondary to vaginitis may have caused superficial epithelium of labia to heal subsequently with fibrous adhesions. Chronic and untreated inflammation creates an environment that causes a tendency to fibrosis and eventual scarring despite normal estrogen levels. The reason why not all of the cases with untreated severe vaginitis develop with labial adhesion is another point of discussion. On the other hand the rapid development of the labial adhesion in a period of only 3 weeks, without any mechanical and traumatic etiology, hormonal abnormality, or underlying dermatological disease in this case, is also dramatic.

The usual solution for cases of adult labial adhesion is surgical adhesiolysis. With labial adhesion in children favorable results were usually obtained with topical estrogen and good hygiene practice. For adult patients, estrogen therapy is not always successful as a first-line treatment and surgery may be necessary [8]. In the present case, because of the tight adhesion of the labia, topical estrogen or manual separation may not have solved the problem. In this case, successful treatment could be carried out with surgical intervention.

In conclusion, we emphasize the possibility of the occurrence of labial adhesions following untreated severe vaginitis in reproductive aged women with normal estrogen levels. It is possible to have labial fusion in patients without any of the known risk factors and more research needs to be conducted to evaluate other etiologies.
